# Inhibitory Effects of Carrageenans on Endotoxin-Induced Inflammation

**DOI:** 10.3390/md18050248

**Published:** 2020-05-10

**Authors:** Irina M. Yermak, Aleksandra V. Volod’ko, Eleonora I. Khasina, Viktoriya N. Davydova, Evgeniy A. Chusovitin, Dmitry L. Goroshko, Anna O. Kravchenko, Tamara F. Solov’eva, Victor V. Maleev

**Affiliations:** 1 G.B. Elyakov Pacific Institute of Bioorganic Chemistry, Far Eastern Branch, Russian Academy of Sciences, Prospect 100 let Vladivostoku 159, Vladivostok 690022, Russia; morskaia@list.ru (A.V.V.); viktoria@piboc.dvo.ru (V.N.D.); kravchenko_25.89@mail.ru (A.O.K.); soltaf@mail.ru (T.F.S.); 2 Federal Scientific Center of the East Asia Terrestrial Biodiversity, Far Eastern Branch, Russian Academy of Sciences, Prospect 100 let Vladivostoku 159, Vladivostok 690022, Russia; eleonorakhas@mail.ru; 3 Institute for Automation and Control Processes, Far Eastern Branch, Russian Academy of Sciences, 5 Radio St., Vladivostok 690041, Russia; eliot@list.ru (E.A.C.); goroshko@iacp.dvo.ru (D.L.G.); 4 Far Eastern Federal University, 8 Sukhanova St., Vladivostok 690950, Russia; 5 Central Research Institute of Epidemiology, Russian Federal Service for Supervision of Consumer Rights Protection and Human Welfare, 3a, Novogireyevskaya St., Moscow 111123, Russia; maleyev@pcr.ru

**Keywords:** carrageenan, lipopolysaccharide, macromolecular structure, nonspecific resistance to lipopolysaccharide, cytokines, enteric infections, salmonellosis

## Abstract

The inhibitory effects of carrageenans (CRGs) on lipopolysaccharide (LPS) induced inflammation in a mouse model of endotoxemia and in complex therapy of patients with enteric infections of Salmonella etiology were studied. The atomic force microscopy (AFM) examination of LPS and its mixture with CRGs showed that the LPS morphology is significantly changed under the action of κ- and κ/β-CRGs. CRGs were able to increase the synthesis of anti-inflammatory interleukin 10 (IL-10) in vitro, and, at low concentrations, their activity in the mixture with LPS was higher. The protective effect of CRGs against *Escherichia coli* LPS was studied in vivo by monitoring the biochemical and pathomorphological parameters. The κ- and κ/β-CRGs and food supplement “Carrageenan-FE” increased the nonspecific resistance of mice to *E. coli* LPS at the expense of the inhibition of processes of thymus involution, adrenals hypertrophy, thyroid atrophy, hypercorticoidism, glycogenolysis, and lactate acidosis. The estimation of the therapeutic action of food supplement Carrageenan-FE in complex therapy of patients with enteric infections of Salmonella etiology is given. Carrageenan-FE restores the system of hemostasis and corrects some biochemical indicators and parameters in the immune systems of patients. These results allow us to hope for the practical application of CRGs for lowering the endotoxemia level in patients under the development of the infectious process caused by Gram-negative bacteria.

## 1. Introduction

The phenomenon of subclinically elevated levels of endotoxin in the bloodstream has recently been termed “metabolic endotoxemia” [1,2]. This elevation is not evident in the clinical setting, but is currently being studied as a significant potential etiology of chronic diseases such as atherosclerosis, type II diabetes mellitus, Parkinson’s disease, pancreatitis, amyotrophic lateral sclerosis, Alzheimer’s disease, and cancer metastasis that arise in the context of chronic low-severity inflammation [1,2,3]. Endotoxins are known to consist of amphiphilic lipopolysaccharide (LPS) macromolecules located on the surface of Gram-negative bacteria. Released from bacteria in vivo or administered in an isolated form, endotoxins exert both physiological and powerful pathophysiological effects in higher organisms and, thus, represent important virulence factors of Gram-negative bacteria [4]. Circulating LPS is bioactive in vivo and correlates with measures of innate and adaptive immune activation [5]. LPS is the primary target for interaction with components of the host immune system, which can be activated by LPS to produce synthesis of tumor necrosis factor-alpha (TNF-α), interleukin (IL)-6, IL-8, and free radicals, such as reactive oxygen species (ROS) [6]. The overexpression of these pro-inflammatory mediators may result in fever, severe damage of tissues, coagulopathy, endothelial dysfunction, vascular instability, apoptosis, multiorgan failure, and septic shock. The gastrointestinal tract is involved in the initial response to the systemic inflammatory reaction [7]. Impaired intestinal barrier function and/or increased epithelial permeability may promote the translocation of bacteria and endotoxin from the gut into the body, increasing the susceptibility to infections [8].

The gut microbiota seems able to promote systemic low-grade inflammation, insulin resistance, and enhanced cardiovascular risk through a mechanism that involves the increased exposure to bacterial products coming from the gut, particularly to the LPS [9,10]. Rich sources of LPS live in the upper respiratory and gastrointestinal tracts, especially in the mouth and colon. LPS may also be found in foodstuffs [11].

Emerging human clinical studies have demonstrated that diet and nutritional supplements have demonstrated the ability to provide clinically important reductions in circulating endotoxins and improve related effects, such as inflammation and other negative health markers [12]. Food fibers are in close contact with the large intestine’s immune system, which composes a significant part of the common immune system. The deficiency of food fibers in the diet promotes the appearance of many gastrointestinal and metabolic diseases [13].

Seaweed polysaccharides, such as carrageenans (CRGs), are one source of soluble dietary fibers [14]. CRGs are sulfated linear galactans, whose basic structural units are disaccharide-carrabiose, consisting of alternating β-1,3-linked and α-1,4-linked galactose residues. Variation in this basic structure results from the 3,6-anhydrogalactose content, location, and number of sulfate groups [15]. The three most industrially exploited types, namely κ-, ι-, and λ-CRG, are distinguished by the presence of one, two, and three ester-sulfate groups per repeating disaccharide unit, respectively. Native carrageenans always represent complex hybrid structures or are generally a mixture of galactans composed of different carrabiose types. The hybrid nature of CRGs at the molecular level is responsible for changes in the biological and physico-chemical properties of CRGs compared with those of their homopolymeric ideal types [16].

Although CRGs have been used as safe food ingredients for a long time, they are also used as a classical model for inducing inflammation. Inflammation induced by carrageenan was originally described by Winter [17]. The CRG-induced edema, as an inflammation model, is usually used to assess the contribution of natural products to resist the biochemical changes associated with acute inflammation. When CRG is injected, acute inflammation with edema appears, along with a production of free radicals as well as a release of the inflammatory mediators [18]. At the same time, intensive studies have shown that CRGs can be regarded not only as foodstuff ingredients, due to a wide spectrum of biological and physiological activities, such as antiviral [19], anticoagulant [20], antitumor [21], and immunomodulatory activities [22]. At present, CRG has been included in the United States Pharmacopeia 35-National Formulary 30S1 (USP35-NF30S1), the British Pharmacopoeia 2012 (BP2012), and the European Pharmacopoeia 7.0 (EP7.0), implying that CRG may have a promising future as a pharmaceutical excipient. According to the JECFA, only degraded carrageenans were associated to adverse effects and should not be used as food additives. It known that degraded low-molecular-weight carrageenan exhibits toxicological properties at high doses, stimulates ulceration of the fecal mucosa, histopathological changes, epithelial thinning, slight erosion, cellular infiltration, and other negative changes in animal organisms [23]. Native high-molecular-weight carrageenan is safe and non-toxic product. The safety of CRGs is supported by a large number of animal oral safety studies in which no adverse effects were reported at high doses (up to 5% in the diet) [24]. The wide range of potential pharmacological applications of different types of CRGs is the main reason for the increased interest in these polysaccharides. The effect of sulfated polysaccharides on metabolic endotoxemia has only been studied in animals. It has been shown that the administration of sulfated polysaccharides to high fat-diet-fed mice increased the amount of short-chain fatty acids in the intestinal tract, decreased the blood LPS or LPS binding protein concentration, and attenuated weight gain [25,26]. The influence of CRG on LPS and on the antibacterial host defense systems, such as the chemotactic responses to heat-killed bacteria or LPS in the pleural cavity and interleukin-1 production by pleural macrophages, was studied by Tatede et al. [27]. Their results demonstrate the potential activity of iota-CRG to enhance antibacterial host defense systems in mice. The administration of κ-CRG has been reported to increase the phagocytic activity of carp, *Cyprinus carpio*, and its resistance against *Edwardsiella tarda* and *Aeromonas hydrophila* administered via intraperitoneal injection [28]. The administration of iota-CRG has been reported to increase the innate immune response of the orange-spotted grouper, *Epinephelus coioides*, and its resistance against *Vibrio alginolyticus* administered via injection [29]. It has been shown that white shrimp, *Litopenaeus vannamei*, that received lambda-CRG exhibited higher immune ability as well as resistance against Gram-negative bacterium *V. alginolyticus* [30].

In a previous study, we showed that the administration of CRGs significantly prolonged the survival of mice against LPS challenge [31]. The degree of protection depended on the structure of the CRGs, their dose, route, and time of administration. We previously demonstrated a potential application of CRGs as protectors against S and R forms of *Proteus mirabilis* LPS. We concluded that CRGs might be considered as anti-endotoxin agents, neutralizing LPS and abolishing their proapoptotic activity [32]. The results obtained in this study by three serological methods indicate the role of *P. mirabilis* lipid A in the binding to different CRGs. The lipid A component is responsible for much LPS toxicity. At the same time, it is known that in the macromolecular mechanism of endotoxin induction many of the observed biological effects of LPS can be related to their physical state, mainly to the three-dimensional supramolecular structure of the LPS assembly [33]. We previously demonstrated that LPS interact with polysaccharide chitosan, which is accompanied by the essential modification of some immunological properties of LPS [34,35]. Later we discovered a transformation of the supramolecular structure of LPS after binding to chitosan [36]. It was necessary to establish whether a change in the macromolecular structure of LPS occurs in the presence of CRG.

In the present study, we examined the effect of CRGs on LPS-induced endotoxemia in mice as well as the influence of the biologically active food supplement “Carrageenan-FE” on the immune system and hemostasis parameters of patients with food borne infections caused by Gram-negative bacteria. To explain the mechanism of the protective effect of CRGs on LPS, the effect of these polysaccharides on the supramolecular structure of LPS was studied.

## 2. Results

### 2.1. Carrageenans

Polysaccharides were extracted from red seaweed, namely *Chondrus armatus* and *Tichocarpus crinitus*, with water at 90 °C. The obtained extracts were purified of low-molecular-weight impurities by column filtration, and polysaccharides were precipitated from solutions by precipitation in alcohol. The yield of polysaccharides from *C. armatus* was 50% (designated as unfractionated or total polysaccharide (Σ)), and the yield from *T. crinitus* was 30%. The polysaccharides were separated using 4% KCl into KCl-insoluble and KCl-soluble fractions. The structures of the polysaccharides were studied by ^13^C-NMR and FTIR spectroscopy. The obtained spectra were compared with the spectra of polysaccharides isolated by us earlier from these species of algae [37,38]. The identity of the spectra indicated that the KCl-insoluble fraction from *C. armatus* was κ-CRG (G4S-DA-carrabiose) with traces of ι-CRG (G4S-DA2S-carrabiose), which were randomly distributed along the polysaccharide chain as a single insertion [37]. The KCl-soluble fraction from *C. armatus* was represented mainly by λ-CRG. According to data obtained by ^13^C-NMR and FTIR spectroscopy, the KCl-insoluble polysaccharides from *T. crinitus* had hybrid structures. On the basis of the analysis of spectra compared with data obtained previously, these polysaccharides were identified as κ/β-CRG [38]. Therefore, CRGs are differentiated by the presence of 3,6-anhydro-D-galactose in κ- and κ/β-CRG as well as by the different numbers and positions of ester sulfate groups. λ-CRG has a higher degree of sulfatation.

The chemical structures of the disaccharide repeating units of the CRGs and viscometric molecular weights of CRGs [39], calculated by the Mark–Kuhn–Houwink equation, are listed in Table 1.

### 2.2. Atomic Force Microscopy of LPS and LPS–GRG Mixtures

**Figure 1:**
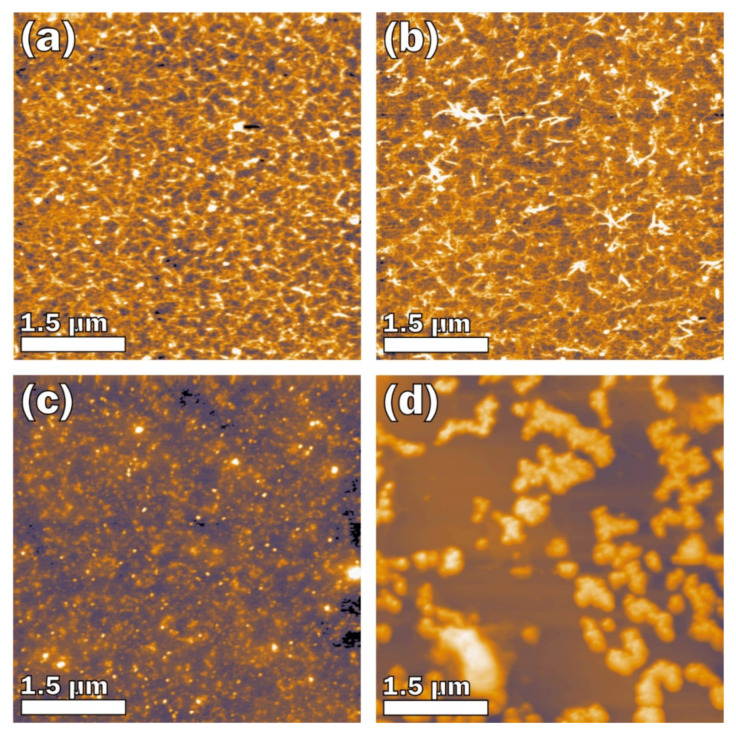
AFM topography images: (**a**) κ-CRG, 0.1 mg/mL; (**b**) κ/β-CRG, 0.1 mg/mL; (**c**) lambda-CRG, 0.1 mg/mL; and (**d**) LPS, 0.05 mg/mL.

The influence of CRGs on the morphology of LPS was investigated by atomic force microscopy (AFM). According to the AFM data, κ-CRG at 0.1 mg/mL (Figure 1a) is a densely branched network structure formed by fibers with a height of about 1.0–1.5 nm and a lateral size of 51 ± 15 nm (root mean square (σRMS) = 0.726 nm). The network structure was also observed for κ/β-CRG at 0.1 mg/mL (Figure 1b). The network is also formed by fibers but they are grouped into bundles and as a result the network looks less dense than the network of κ-CRG. These fibers have a height of about 0.9–1.5 nm, with a thickness of 36 ± 11 nm (σRMS = 0.705 nm). λ-CRG differs from the other investigated carrageenan types with a high sulfation degree and the absence of 3,6- anhydrogalactose. λ-CRG is presented in the form of a random coil (Figure 1c). According to the AFM data, λ-CRG at 0.1 mg/mL forms an unordered structure consisting of particles with an irregular spherical segment (σRMS = 0.951 nm). The density of the particles is about 1.1 × 1010 cm^−1^, they are distributed almost uniformly over the surface, but there are a number of particle agglomerations. The height and lateral size of the particles are about 1.8–2.0 nm and 38 ± 9 nm, respectively.

The AFM image of *E. coli* LPS shows micelle-like particles typical for amphiphilic polymers (σRMS = 25.859 nm). The large particles easily seen in Figure 1d have a density of 2.6 × 108 cm^−2^ and they are nonuniformly distributed over the sample surface. The average large particle height is about 50.9 nm. Most particles have an elongated irregular shape with a large lateral size of 651 ± 452 nm and a small lateral size of 334 ± 115 nm; the aspect ratio is about 2. A detailed analysis of the large particles revealed that they consist of small particles, of which the lateral size can only be roughly estimated to be about 40 nm because of a lack of resolution.

**Figure 2:**
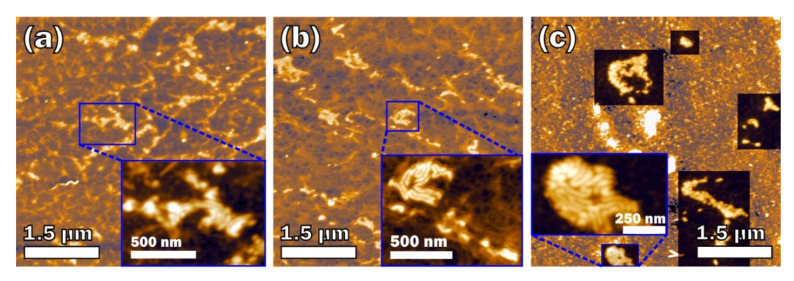
AFM topography images of mixtures in ratio 2:1 *w*/*w*: (**a**) κ-CRG + LPS; (**b**) κ/β-CRG + LPS; and (**c**) λ-CRG + LPS.

The image of the *E. coli* LPS with CRG mixtures at the 2:1 *w*/*w* ratio between components (Figure 2) showed that the macromolecular structures of the mixtures are quite different from the structures of the initial components at the same concentrations. In the case of κ-CRG and κ/β-CRG, the number of separate fibers is significantly decreased. In the complex κ-CRG + LPS, one can see fragments of the network with many spherical particles as can be seen in clean *E. coli* LPS (Figure 2a), and some worm-like structures (Figure 2a, inset). The lateral size and height of the spherical particles are 35 ± 3 and 1.65 ± 0.60 nm, respectively, while the width and height of the fibers in the worm-like structures are 60 ± 18 and 7.0 ± 1.1 nm, respectively. The fibers in the κ/β-CRG + LPS mixture are organized in a continuous network with round-shaped holes (Figure 2b), while the worm-like structures are either incorporated into the network or just lie over it. The width and height of the fibers in the worm-like structures are 49 ± 11 and 6.6 ± 1.4 nm, respectively, which are slightly smaller than the corresponding values in the κ-CRG + LPS mixture. The value of σRMS for both mixtures (Figure 2a,b) increased to more than twice that for the initial CRGs: 2.594 nm for κ-CRG + LPS and 1.989 nm for κ/β-CRG + LPS.

The morphology of λ-CRG in the complex with *E. coli* LPS also changed (Figure 2c). Instead of separated particles, one can see short fibers that are uniformly distributed over the surface rather than arranged in a network. The width and height of the fibers are 27 ± 3 and 0.74 ± 0.15 nm, respectively. *E. coli* LPS in this mixture also forms the worm-like structures that are incorporated into a layer of uniformly distributed fibers of λ-CRG. The width and height of the fibers in the worm-like structures are 62 ± 13 and 8.6 ± 2.0 nm, respectively.

### 2.3. Effect of LPS and CRGs on the Production of Cytokines by Human Cells

Experiments were performed to determine the effect of CRGs on the activation of the synthesis of LPS-induced IL-10 and TNF-α in human blood cells.

**Figure 3:**
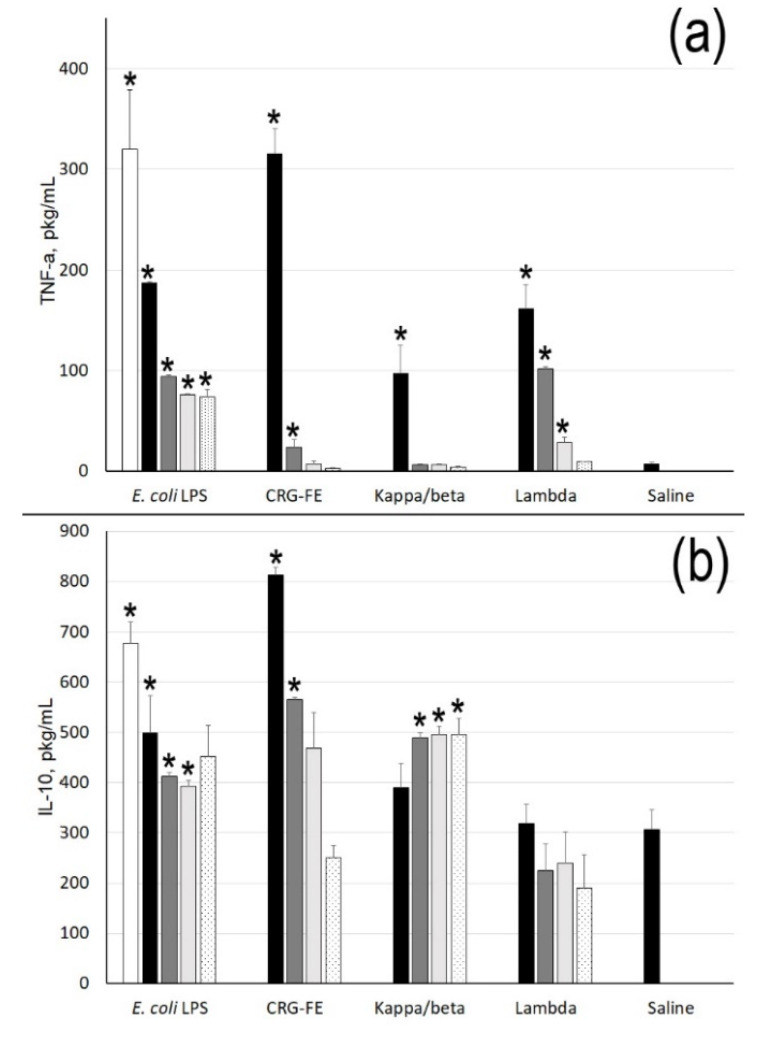
TNF-α (**a**) and IL-10 (**b**) levels stimulated by *E. coli* LPS and carrageenans. Concentrations of samples: 10 µg/mL (white column), 1 µg/mL (black), 100 ng/mL (gray), 10 ng/mL (light gray), and 1 ng/mL (black-dot). Mean (±SD) contents of cytokine in serum are presented. Whole blood was obtained from five healthy subjects and incubated with the samples at different concentrations. The level of cytokines in serum of normal donors was considered as a negative control used for statistical significance calculation. * Differences between the samples and the control were significant, *p* < 0.05.

As shown in Figure 3a, CRGs induced the synthesis of TNF in cells only at high polysaccharide concentrations. Σ-CRG at C = 1 µg/mL showed a high activity with respect to the synthesis of the pro-inflammatory cytokine, similar to the effect of LPS at C = 10 µg/mL. At the same time, CRGs induced the synthesis of IL-10 in a dose-dependent manner similar to LPS. κ/β-CRG can induce IL-10 synthesis and is weakly dependent on the polysaccharide concentration.

**Figure 4:**
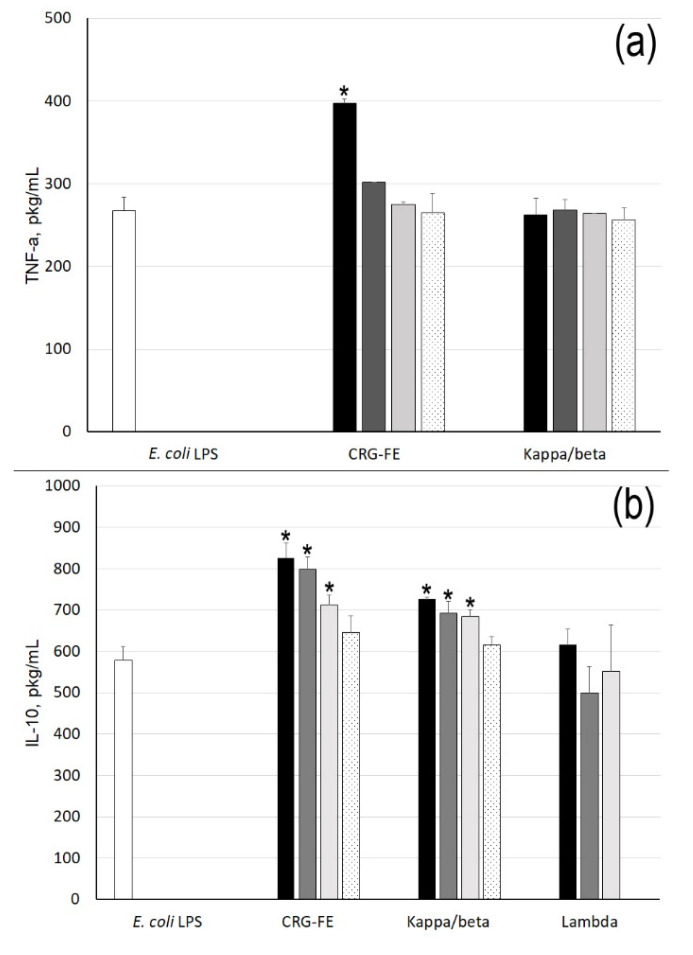
TNF-α (**a**) and IL-10 (**b**) levels stimulated by preliminary incubation cells with *E. coli* LPS (10 min), and then CRGs. Concentrations of samples: 10 µg/mL (white column), 1 µg/mL (black); 100 ng/mL (gray); 10 ng/mL (light gray), and 1 ng/mL (black-dot). Mean (±SD) contents of cytokine in serum are presented. The level of cytokines in serum stimulated by *E. coli* LPS (10 µg/mL) was considered as a control used for statistical significance calculation. * Differences between samples and the control were significant, *p* < 0.05.

The preliminary incubation of cells with LPS (C = 10 µg/mL) followed by treatment with λ-CRG did not have an influence on IL-10 production, whereas treatment with κ- and κ/β-CRG (Figure 4) increased the production of IL-10 and slightly reduced the synthesis of TNF in cells.

### 2.4. Effect of CRGs on Stress Reaction of Mice Induced with E. coli LPS

When considering the effect of *E. coli* LPS injection for five days, it was found that the physiological status of animals exhibits considerable changes. The stress response to LPS- intoxication in mice is manifested by thymus involution, thyroid atrophy, and adrenal gland hypertrophy, at the same time as an increase of the serum corticosterone level, a decrease of the concentrations of adenosine triphosphate (ATP) and glycogen in liver, and lactate acidosis (Table 2).

The results of this study show that administration of κ-CRG, κ/β–CRG, and ∑-CRG led to the minimization of physiological disorders and metabolic homeostasis in mice exposed to LPS- intoxication. The relative masses of the thymus, thyroid, and adrenal glands of animals in the LPS + ∑-CRG group differed from the norm only by 22%, 10%, and 12%, respectively, whereas, in the mice of LPS group, the deviations were 36%, 21%, and 30%, respectively. In the LPS + κ/β-CRG group, the relative masses of the thymus, thyroid and adrenals were less than in the control group by 16%, 7% and 5%, respectively (Table 2). After the administration of the κ- and κ/β-CRGs, the serum corticosterone level in mice intoxicated with *E. coli* LPS were reduced by 32% relative to the LPS group. The administration of ∑-CRG and κ/β-CRG led to 22% and 17% lowered lactate storage in mice liver relative to the LPS group. The use of κ-CRG, κ/β-CRG, and ∑-CRG contributed to more effective conservation of the energetic substrate ATP and glycogen in liver. The concentration of ATP and glycogen in mice liver were on average 18% higher than in the LPS group.

In this study, κ-CRG significantly minimized adrenal gland hypertrophy, and the depletion of ATP and glycogen in mice liver, whereas λ-CRG did not have an effect on the stress response of mice exposed to *E. coli* LPS.

### 2.5. The Effects of Food Supplement Carrageenan-FE on the Immune System and Hemostasis Parameters in Patients with Food Borne Toxicoinfection

The medico-biological study was carried out in accordance with The Code of Ethics of the World Medical Association (Declaration of Helsinki) and the biologically active food supplement Carrageenan-FE based on the ∑- CRG, was used.

Investigation of the parameters of the hemostasis system showed that 18 patients with food borne toxicoinfection, caused by *Salmonella enterica*, showed signs of a hypercoagulation (first phase of disseminated intravascular coagulation) and 24 patients showed symptoms of a hypocoagulation (second phase syndrome). For patients with hypercoagulation, there was a reduction of platelet aggregation by an average of 65%, and, in patients with hypocoagulation, the degree of aggregation increased on average by 22%. In patients on the third day of treatment with CRG, there was a decrease of leukocytosis, decreasing to normal amounts of activated immune cells, and an increase in relation to the total population of T-lymphocytes and the absolute number of lymphocytes in peripheral blood and their immunoregulatory subpopulations (Table 3).

## 3. Discussion

The emergence of multi-drug resistant Gram-negative bacteria “super bugs” are becoming a serious therapeutic problem. Animal and human studies indicate LPS as an antigen that activates the immune system, playing an important role in the pathogenesis of metabolic chronic diseases [11]. The application of bactericidal antibiotics, apart from killing the bacteria, may lead to the massive release of endotoxin. The search for non-toxic effective endotoxin binding and neutralizing compounds is an ongoing process.

One of the promising ways to inhibit the harmful inflammatory responses of endotoxins is to bind LPS with certain polycations, which can interact with lipid A as a result, blocking this toxic center of the endotoxin. In recent years, it has been demonstrated that antimicrobial peptides from the Pep 19-2.5 family, which were designed to bind to LPS, act as anti-inflammatory agents against sepsis and endotoxic shock caused by severe bacterial infections. The peptide-mediated neutralization of LPS and LP involves changes in various physical parameters, including both the gel to liquid crystalline phase transition of the acyl chains and the three-dimensional aggregate structures of the LPS [40]. It is known that LPS as amphiphilic molecules tend to form supramolecular aggregates in aqueous solutions at concentrations above the critical micellar concentration. LPS molecules may aggregate into different physical structures, including micelles, inverted micelles, or bilayers and undergo lamellar to inverted hexagonal or cubic phase transitions depending on the physiochemical environment [41]. The complex hydrodynamic geometry exhibited by LPS in dilute suspensions may have consequences for the interpretation of LPS biological activity in the host immune response [42].

As shown by our previously obtained data using dynamic light scattering, CRGs changed the surface charge and the particle size of LPS. This process depended on the structural type of carrageenan, the initial concentration of the components, and their proportion in the solution [43]. In the current work, AFM was used to study the probable modification of LPS morphology due to the effect of CRGs. Compared with other microscopic methods, AFM is characterized by minimal artifacts related to the fixation and staining of samples. Furthermore, the height and width of objects on AFM images may provide additional information about the degree of association and polymer heterogeneity. The macromolecular structures of LPS with κ-, κ/β-, and λ-CRGs were investigated. Comparative AFM examination of LPS and its mixture with CRGs showed that LPS morphology is significantly changed under the action of the polysaccharide. The macromolecular structure of LPS, determined using AFM, shows a change in LPS aggregates of the micellar structure into worm-shaped formations in the presence of CRGs. In the case of κ- and κ/β-CRGs, the number of separate fibers of CRG is significantly decreased. The AFM images show that the LPS vermicular structure is integrated into a three-dimensional network of κ- and κ/β-CRGs (incorporated into the network or just lying over it). In a mixture with λ-CRG, a change in the LPS macromolecular structure is also observed (Figure 2c). However, in this case, the absence of the three-dimensional structure of λ-CRG does not limit the assembly of these vermiform LPS particles into larger associates. It is worth nothing that the worm-like structures form the densest agglomerates in λ-CRG. We suppose that the worm-like structures consist of LPS, which has changed its morphology under the influence of the CRG network consistent with the electron microscopy data we obtained earlier [43].

It is known that, when entering the bloodstream during local or systemic Gram-negative infections, endotoxin has an impact on almost all the systems of an organism, causing a number of pathophysiological changes [44]. Recently, in a LPS-induced endotoxemia mouse model, it has been shown that hirsutanol A (isolated from the red-algae-derived marine fungus *Chondrostereum* sp.) pretreatment improved endotoxemia-induced acute sickness behavior, including acute motor deficits and anxiety-like behavior [45].

A nonspecific resistance of the organism to *E. coli* LPS induced by CRG was studied in the present work. We investigated the effect of different structural types of CRGs on the LPS-induced intoxication of animals by the degree of variability of biochemical and pathological parameters to those most adequately responding to any valid stressor, including bacterial endotoxin. In our experiments, LPS parenterally injected into mice (in a nonlethal dose) caused significant changes of these parameters. Preventive oral administration of CRGs significantly reduced the morphological, endocrine, and metabolic disorders caused by endotoxin in the liver, thymus, spleen, adrenal glands, and blood of animals (Table 2). The results of these studies have shown that biochemical and pathomorphological manifestations of endotoxemia induced by intraperitoneal injection of bacterial LPS were less pronounced in the mice that received CRGs. ∑-, κ-, and κ/β-CRGs increase the resistance of the host organism to bacterial LPS. As seen in these experiments, these CRGs are more active than λ-CRG, which has a high degree of sulfation and does not form a three-dimensional structure. As shown by the AFM data (Figure 2), LPS is embedded in the three-dimensional structure of κ- and κ/β-CRGs, which probably contributes to a greater screening of its toxicity. Moreover, as we recently showed, κ- and κ/β-CRGs have the most powerful mucoadhesive properties, which may prevent LPS from landing on the epithelial layer [46].

Our data are consistent with results that demonstrate how fucoidan prevented endotoxin-induced damage in a mouse model of endotoxemia and increased the mice’s resistance to LPS. The parenteral or per os administration of fucoidan resulted in decreasing the degree of microcirculatory disorders and secondary dystrophic-destructive changes in parenchymal organs of mice with endotoxemia [47].

Although the mechanisms responsible for the CRG effects require further study, the data obtained provide strong evidence for a normalizing effect of CRGs on the state of organs of mice with endotoxemia. The CRG-induced host resistance to endotoxin may result from a variety of reasons, one of which may be associated with a change in the macromolecular structure of LPS. The resistance to endotoxin induced by CRGs can be attributed to the immunomodulating effect of polysaccharide. As is known, CRGs and endotoxins also stimulate the biosynthesis of different mediators of the immune system, such as IL-10, IL-8, IL-6, and TNF-α [6,44]. Since cytokines play a critical role in regulating inflammatory and immunological processes of the host, in vivo administration of CRGs may influence antibacterial host-defense systems. It is known that IL-10 has great potential for use in the treatment of inflammatory and immune illnesses. It has been shown that IL-10 protects mice against lethal doses of endotoxin [48]. In addition, IL-10 treatment inhibits the activation of cytokine synthesis during experimental endotoxemia in primates [49] and humans [50] and in microglia cell cultures [51].

Previously we have shown [22] that CRGs of different structural types induced the synthesis of the anti-inflammatory cytokine IL-10 in human blood cells, which rose with an increase in polysaccharide concentration. κ/β-CRG showed fairly high activity independent of the concentration. κ/β-CRG under oral administration into mice possesses a protective effect and reduces an intensity of inflammatory response induced by LPS probably due to some modulating effect on the cellular activity of peritoneal leukocytes and to a greater degree on cytokine production [52].

In the present work, κ/β-CRG caused an increase in the level of the cytokine IL-10 in blood compared with the control (Figure 3). The effect of ∑-CRG on the induction of IL-10 synthesis exceeded the LPS effect at the same concentration by almost more than 1.5 times. Along with this, ∑-CRGs and κ/β-CRG at concentrations of 1–100 ng/mL showed weak activity to stimulate the synthesis of pro-inflammatory cytokine TNF-α. These polysaccharides also enhanced the effect of LPS, increasing the synthesis of the anti-inflammatory cytokine IL-10 in a test with the pre-treatment of blood cells with *E. coli* LPS. λ-CRG showed a different effect on the stimulation of cytokine synthesis in cells. This polysaccharide did not have the ability to stimulate the synthesis of anti-inflammatory cytokine IL-10, but it possessed an adequate ability to induce pro-inflammatory cytokine synthesis increasing the level of TNF-α in serum at 1 μg/mL and 100 ng/mL, which was comparable to the effect of LPS at the same concentrations.

Known disorders of coagulation or disseminated intravascular coagulation (DIC) induced by endotoxin lead to multiple-organ dysfunction [53]. An important role in the development of DIC has been shown to belong to platelets and LPS-induced platelet aggregation [54]. Earlier in the in vitro experiment, we showed that CRGs significantly inhibited LPS-induced upregulation of reactive oxygen species reduced or completely inhibited collagen-induced platelet aggregation and decreased their aggregation activity caused by the cooperative effect of LPS and collagen [43]. In the clinical study, we showed the effects of food supplement Carrageenan-FE on the immune system and lipid profile in patients with cardiovascular disease. Carrageenan-FE moderately modulated all of the immunity system markers and caused statistically significant decreases in the biomarkers of chronic inflammation [55].

It is known that the overreaction of the immune system into fragments of bacterial cells, such as LPS, underlies many inflammatory bowel diseases. In this regard, in the complex treatment of intestinal infections, the use of agents that restore normal microflora is indicated [56]. In the current study, we investigated the therapeutic effect of CRG in complex therapy of patients with enteric infections of Salmonella etiology. Our results show that the application of food supplement Carrageenan-FE in the standard therapy of toxicoinfections contributes to the correction of hemostasis. In this case, the action of CRG had a modulating regulatory pattern: in patients with hypercoagulation, a decrease in platelet aggregation activity was observed, and, in patients with hypocoagulation, the degree of aggregation increased. The application of Carrageenan-FE in the standard therapy scheme corrected some biochemical indicators and parameters of the immune system of the patient organism more actively in comparison with a control. A quick recovery of the immune system of patients taking CRG is likely due to its immunoregulatory properties.

These results allow us to hope for the practical application of CRGs for lowering the endotoxemia level in patients under the development of the infectious process caused by Gram-negative bacteria.

## 4. Materials and Methods

### 4.1. Polysaccharides

Algae: The following species of red algae were collected at The Peter the Great Bay, Japan Sea, which is near the border between the boreal and tropical zones: *Chondrus armatus* (Gigartinales, Gigartinaceae) and *Tichocarpus crinitus* (S.G. Gmelin) (Gigartinales, Tichocarpoaceae). All algae were harvested at the end of August and identified by Prof. E. Titlynov and T. Titlynova (National Scientific Center of Marine Biology, Far-Eastern Branch of the Russian Academy of Sciences). The selected seaweeds were in the vegetative form lacking any reproductive organs. The algae were washed with tap water to remove excess of salt. Bleaching of the seaweed was by maintaining the specimen in pure acetone for 3 days prior being dried in the air.

Extraction of CRGs: Dried and milled algae (50 g) were suspended in hot water (1.5 h) and the polysaccharides were extracted at 90 °C for 2 h in a water bath. The residue was removed by centrifugation and supernatant poured into ethanol (3 volumes), yielding the crude extract (unfractionated) of polysaccharides. The crude extracts were purified by redissolving in water, concentrated, dialyzed, and freeze-dried, yielding Σ-CRG. Then, the polysaccharides were separated into gelling KCl-insoluble and non-gelling KC1-soluble fractions as described previously [34] and their structures were established according to the published protocol. λ- and k-CRGs from *C. armatus* and κ/β-CRG from *T. crinitus* were obtained. The biologically active food supplement Carrageenan-FE (Pacific Institute of Bioorganic Chemistry Far East Branch of the Russian Academy of Sciences) is composed of κ- and λ-CRGs from *C. armatus* at a ratio 3:1 (*v*/*v*) and is labeled as Ʃ-CRG. The endotoxin admixture in samples of CRGs was determined by the fluorogenic endotoxin detection assay PyroGene rFC purchased from Lonza (USA) in accordance with test instruction. The level of environmental endotoxin was low: 0.60 EU mL^−1^ in doses of 100 mg/mL.

Commercial LPS from *Escherichia coli* 055:B5 (Cat No: L2880, Lot No: 102M4017V, Sigma, St. Louis, MO, USA) was used in the study.

Molecular Weight Estimation: Viscosimetric molecular weight of the polysaccharide sample was calculated using the Mark–Kuhn–Houwink equation: |η| = KmMα, where |η| is the intrinsic viscosity and Km and α are empirical constants for carrageenan constituting 3 × 10^−3^ and 0.95 at 25 °C in 0.1 M NaCl, respectively, according to the literature data for this polymer–solvent system [36]. The viscosity of polysaccharide solution (1–2 mg/mL in 0.1 M NaCl) was measured with a modified Ubbellohde viscometer (Design Bureau Puschino, Russia) with a capillary diameter of 0.3 mm at 25 °C, the time of accuracy being within ±0.1 s. The intrinsic viscosity of the CRGs sample was calculated by the extrapolation of the dependence ln (η)rel/C to infinite dilution using the least squares method.

### 4.2. Atomic Force Microscopy (AFM)

LPS was dissolved in distilled de-ionized water at concentration of 0.05 mg/mL; CRG samples were at concentrations of 0.1 mg/mL. The LPS–CRG mixture were prepared at the same concentrations. The CRG solution was mixed with LPS solution (2:1 *w*/*w*). Aliquots (12 µL) of the aqueous solutions of complex and their initial component were deposited onto freshly cleaved mica and dried at 37 °C for 24 h or at 70 °C for 30 s (for LPS). The morphology of CRG, LPS, and their mixtures was studied in air by AFM (Solver P47) in the tapping contact mode using a tip with the radius of 10 nm.

### 4.3. IL-10 and TNF-α Inducing Activity on the Human Blood Cells

The blood processing was performed using procedure of De Groote et al. Peripheral blood was collected by vena puncture into sterile siliconized tubes containing 30 IU of lithium heparinate per 5 mL tube diluted 1:5 in sterile Medium 199 (Sigma-Aldrich, Saint Louis, MO, USA) containing 300 mg/L of glutamine (Gibco, Life Technology, Darmstadt, Germany) and 50 µg/mL of gentamicin. Diluted blood (0.1 mL) was transferred into sterile polypropylene plates and then incubated with the LPS, carrageenans, or with LPS and carrageenan (370 °C, 5% CO_2_). After 24 h, the supernatants were collected and frozen followed by cytokine determination using a specific ELISA kit (“Cytokine”, Saint-Petersburg, Russia). The study protocol was approved by the medical ethics committee of the local hospital (Vladivostok, Russia). Informed consent was obtained from all subjects who participated in the study. All donors were free of medicine administration for 14 days prior to blood sampling. Blood was drawn from the antecubital vein of normal healthy human volunteers and anticoagulated in plastic tubes (Greiner Bio-One International AG, Kremsmuenster, Austria) with 30 IU lithium heparinate used as an anticoagulant.

### 4.4. Animals and Diets

The work was carried out in accordance with “Directives 2010/63/EU of the European Parliament and the Council of the European Union for the Protection of Animals used for Scientific Goals” and approved by the Federal Scientific Center of biodiversity FEB RAS Animal Care and Use Committee.

Mature male CD-1 mice were obtained from G.B. Elyakov Pacific Institute of Bioorganic Chemistry, FAR RAS (Vladivostok, Russia). The mice with body mass of 22–24 g kept in standard conditions of the vivarium at a controlled temperature 20–22 °C and ambient humidity 60–65%. Light were maintained on an artificial 12 h light–dark cycle. Each experimental group consisted of eight animals, each mouse in the cage had a floor area of 70 cm^2^, which corresponds to international standards. The mice were provided with water standard feed compliant GOST R 50258-92 (CJSC ProKorm, Russia) ad libitum.

### 4.5. Experimental Design

This part of the study consisted of estimating the influence of different types of carrageenans on the physiological state of mice intoxicated *E. coli* LPS.

The mice were randomly allocated into six groups. All animals except the control group were given intraperitoneally LPS solution in dose 1 mg/kg body mass (0.1 mg/mL, pH 7.0) for five days. The control group received intraperitoneally 0.2 mL saline. The mice of the control and LPS groups were given only standard feed, whereas mice of other groups daily administrated CRGs suspensions in dose 100 mg/kg body mass (4 mg/mL, pH 7.0) 1 h before LPS injection through gastric gavage. At the end of experiment, mice were killed by decapitation, and their inner organs were removed and weighed. The blood samples were centrifuged at 1.200× *g* for 15 min. Relative organ masses of liver, thymus, thyroid, and adrenals were calculated as organ mass (mg)/body mass (g). Serum corticosterone concentrations were determined by the fluorometric method [57]. The glycogen content in liver was estimated with the anthrone reagent [58]. The adenosine triphosphate (ATP) and lactate levels in liver were measured enzymatic spectrophotometric methods used test-system with nicotinamide coenzymes NADF and NAD•H, respectively [59,60]. Statistical analysis was performed using Student’s t-criterion for unpaired data, and p-values of less than 0.05 were considered significant. All data presented as mean ± standard deviation.

### 4.6. Medico-Biological Study of Food Supplement Carrageenan-FE

Ethical approval: The medico-biological study was carried out in accordance with The Code of Ethics of the World Medical Association (Declaration of Helsinki). Informed consent was obtained from all subjects who participated in the study.

The medical-clinical study of food supplementation Carrageenan-FE was carried out on the basis of the permission of the State Committee for Standardization of the Russian Federation (No.035/002158) and the consent of the ethics committee of the Central Research Institute of Epidemiology of the Federal Service on Customers Rights protection and human Well-being Surveillance (Moscow, Russia).

The food supplement Carrageenan-FE (Pacific Institute of Bioorganic chemistry, Far East Branch of the Russian Academy of Sciences, Vladivostok, Russia) is composed of two structural types of CRG. The supplement meets the requirements for food supplements and is recommended as an additional source of food fiber.

Patients diagnosed with enteric infection diseases and healthy volunteers were selected for current clinical trial based on an analysis out-patient medical records obtained from Ethical Committee in the framework of the Central Research Institute of Epidemiology of the Federal Service on Customers Rights Protection and Human Well-being Surveillance. After the patients were selected, they were enrolled in the current clinical trial to test the effects of the CRG-food supplement. The study of the therapeutic effect of CRG-FE in patients with enteric infection diseases with *Salmonella* etiology was carried out at a clinical infectious hospital (Moscow, Russia).

Dynamic examination of 42 patients (men and women, average age 31 years) with severe intoxication syndrome was conducted. Patients came to the hospital for 1–3 days with signs of intoxication: body temperature of up to 38–39 °C, chills, headache, frequent (10–15 times per day) loose stools, nausea, and repeated vomiting. Patients (22) received per os 150 mg of Carrageenan-FE administered together with 150 mL of Ringer solution to three times per day. In the second group, patients received 150 mL of the same solution (standard therapy). In the control group were healthy patients. The results were evaluated by clinical data and research parameters of hemostasis and immunological status. Hemostasis was characterized by the following parameters: prothrombin time, thrombin time, activated partial thromboplastin time, fibrinogen level, and platelet aggregation activity. To assess the state of immune system, relative and absolute number of lymphocytes, T lymphocytes (CD3+), and B-lymphocytes (CD19+), as well as immunoregulatory subpopulation of T-cells helpers (CD4+) and T cytotoxic lymphocytes (CD8+) were calculated. Investigations were carried out by flower cytometer EPICS XL (Beckman Coulter) using monoclonal antibodies IO-Test (double label). Functional activity of venous blood neutrophils was determined via spontaneous and stimulated with the test NBT (NBT-test) and expressed as conventional units.

### 4.7. Statistical Analysis

All measurements were done in three replicates. All results are expressed as mean ± the standard deviation compared by ANOVA. All differences were considered to be statistically significant if *p* < 0.05. Data were analyzed using the software Statistic 6.0. (StastSoft, Tulsa, OK, USA). To confirm the normal distribution of variables, Shapiro–Wilks test was performed.

## 5. Conclusions

In the present investigation, we revisited the effect of different types of CRGs on the LPS-induced intoxication of animals by the degree of variability of biochemical and pathological parameters to those most adequately responding to any valid stressor, including bacterial endotoxin. The data demonstrate that endotoxemia induced by intraperitoneal injection of bacterial LPS was less pronounced in the mice that received CRGs. ∑-, κ- and κ/β-CRGs increasing the resistance of the host organism to bacterial LPS are more active than λ-CRG, which has a high degree of sulfation and does not form a three-dimensional structure. LPS morphology is significantly changed under the action of the polysaccharide. According to AFM data, LPS is embedded in the three-dimensional structure of κ- and κ/β-CRGs, which probably contributes to a greater screening of its toxicity. The host resistance to endotoxin, induced by CRGs, can be caused by its effect on the macromolecular structure of LPS as well as the modulation of cytokine production of the organism by polysaccharide action.

We investigated the therapeutic effect of CRG in complex therapy of patients with enteric infections of Salmonella etiology. Our results show that the application of food supplement Carrageenan-FE in the standard therapy of toxicoinfections contributes to the correction of hemostasis and corrected some biochemical indicators and parameters of the immune system of the patient organism more actively in comparison with a control.

The results of our study reveal the potential application of CRG for treatment of Gram-negative infections associated with the accumulation of endotoxin in an organism.

## Figures and Tables

**Table 1:** Chemical structures of the repeating units of CRG from algae belonging to families Gigartinaceae and Tichocarpaceae.

Source of Carrageenans	Type of CRG	Structure of Disaccharide Repeating Unit	Mw, kDa
*C. armatus* Gigartinaceae	Kappa (κ)	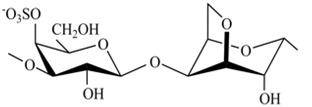	560
	Lambda (λ)	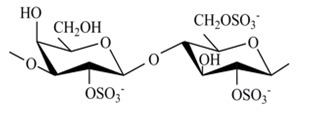	185
*T. crinitus* Tichocarpaceae	Kappa/beta (κ/β) (60:40)	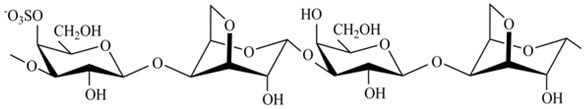	172

**Table 2:** The effect of different types of CRG on some physiological parameters in mice intoxicated with *E. coli* LPS.

**Groups**	**Relative Organ Masses, mg/g BM**			
	**Liver**	**Thymus**	**Thyroid**	**Adrenals**
control	54.16 ± 6.60	1.98 ± 0.22	0.175 ± 0.016	0.217 ± 0.018
LPS	62.79 ± 7.83 ^a^	1.27 ± 0.16 ^a^	0.138 ± 0.018 ^a^	0.282 ± 0.032 ^a^
LPS + ∑-CRG.	60.06 ± 4.77	1.55 ± 0.12 ^b^	0.157 ± 0.013 ^b^	0.243 ± 0.033 ^b^
LPS + κ/β-CRG	61.48 ± 5.74	1.67 ± 0.16 ^b^	0.162 ± 0.017 ^b^	0.228 ± 0.031 ^b^
LPS + κ-CRG	55.25 ± 5.85 ^b^	1.43 ± 0.22	0.148 ± 0.039	0.254 ± 0.014 ^b^
LPS + λ-CRG	60.14 ± 4.96	1.29 ± 0.17	0.153 ± 0.032	0.265 ± 0.029
**Biochemical parameters**				
**Groups**	**Serum corticosterone, μmol/L**	**Liver ATP,** **μmol/g**	**Liver lactate,** **μmol/g**	**Liver glycogen,** **μmol/g**
control	0.28 ± 0.043	2.72 ± 0.222	1.88 ± 0.443	232.6 ± 43.89
LPS	0.39 ± 0.047 ^a^	2.00 ± 0.377 ^a^	2.53 ± 0.350 ^a^	176.6 ± 17.33 ^a^
LPS + ∑-CRG	0.30 ± 0.047 ^b^	2.45 ± 0.337 ^b^	2.10 ± 0.395 ^b^	220.4 ± 41.04 ^b^
LPS + κ/β-CRG	0.30 ± 0.071 ^b^	2.31 ± 0.140 ^b^	2.21 ± 0.217 ^b^	210.6 ± 20.46 ^b^
LPS + κ-CRG	0.33 ± 0.080	2.52 ± 0.338 ^b^	2.44 ± 0.308	230.8 ± 30.22 ^b^
LPS + λ-CRG	0.38 ± 0.047	2.06 ± 0.076	2.60 ± 0.359	185.6 ± 35.82

BM, body mass; ATP, adenosine triphosphate; mean ± SD (n = 8 observations); ^a^ *p* < 0.05 compared with the control group; ^b^ *p* < 0.05 compared with the LPS group used Student’s *t*-test.

**Table 3:** The average values of immunological parameters in patients with acute enteric infections in the course of the disease with treatment Carrageenan-FE.

Indicator		On Admission to the Hospital N = 42	3- Days of Therapy		Control N = 20
			With CRG-FE N = 22	Without CRG-FE N = 20	
Leukocytes * 109/L, abs		8.3 ± 3.3	6.6 ± 2.9^ †‡^	5.7 ± 1.8	6.45 ± 1.5
neutrophils,	%	81.4 ± 8.5 *	60.8 ± 8.9 ^‡^	67.8 ± 5.7	62.5 ± 11.4
	abs × 109/L	6.8 ± 3.2 *	4.2 ± 2.7 ^‡^	3.9 ± 1.3	4.12 ± 1.6
lymphocytes	%	13.7 ± 6.9 *	31.5 ± 7.5 ^†‡^	26 ± 5.4 *	34.0 ± 10.6
	abs × 109/L	1.0 ± 0.4 *	2.0 ± 0.7 ^†‡^	1.4 ± 0.3 *	2.1 ± 0.6
CD3+	%	66.4 ± 8.4	73.5 ± 7.4 ^‡^	74.2 ± 5.2	68.7 ± 7.3
	abs × 109/L	0.7 ± 0.3 *	1.4 ± 0.5 ^†‡^	1.0 ± 0.2 *	1.42 ± 0.3
CD4+	%	43.0 ± 20.0	45.7 ± 7.4	49.8 ± 4.1 *	40.0 ± 17.0
	abs × 109/L	0. 5 ± 0.2 *	0.9 ± 0.3 ^‡^	0.76 ± 0.2	0.81 ± 0.1
CD8+	%	22.3 ± 14.5	24.5 ± 6.4	22.8 ± 5.2	25.6 ± 6.0
	abs × 109/L	0.3 ± 0.1 *	0.5 ± 0.2	0.4 ± 0.2	0.6 ± 0.2
CD4+/CD8+		2.2 (0.4–4.8)	2.0 (0.9–3.6)	2.4 (1.5–3.4)	1.6 (1.1–2.5)
CD 19 %	%	15.4 ± 5.1	11.7 ± 3.4 ^‡^	10.9 ± 4.0	12.4 ± 3.5
	abs × 109/L	0.2 ± 0.1 *	0.2 ± 0.1 ^†‡^	0.2 ± 0.1 *	0.3 ± 0.1

mean ± SD (n, see Table 3); ^*^ *p* < 0.05 compared with the control group; † *p* < 0.05 compared with the without CRG-FE group; ‡ *p* < 0.05 compared with the on admission to hospital group used Student’s *t*-test.
